# Puerperal Eclampsia

**Published:** 1888-11

**Authors:** Wm. P. Fleming

**Affiliations:** Georgetown, Texas


### PUERPERAL ECLAMPSIA.

By Wm. P. Fleming, M. D., Georgetown, Texas.

Read before the Austin District Medical Society, September 20, 1888.

NO MORE fruitful topic for research, and hardly one more difficult, could be proposed than eclampsia. As respects etiology, eclampsia is the most theoretical of all diseases, derivatively speaking, the term is applicable to but a single one of the many phenomena which it is made to cover ; usage has also restricted it to such phenomena occurring in women during puerperium.

Puerperal eclamsia has, by different writers, been divided into several varieties, as hysterical, epileptic, appoplectic, anæmic and peculiar puerperal convulsions; to the latter the term eclampsia having been applied. High authority denies the practicability of this division. The apoplectic is always accompanied by serous infiltration of the tissues and œdema, with symptoms of toxæmia, from absorption of uric acid ; temperature 101 to 105. The patient is always attacked before, or during, and never after labor.

The hysteric form is always preceded by hemicrania, and is more frequently the result of mental excitement, anxiety and depression. The attack occurs during or soon after confinement.

In the anæmic the secretions are normal ; no œdema, temperature generally below normal ; yet this form is attended by the highest mortality.

Modern pathology has modified the ideas of the profession. Cerebral congestion seemed at one time the accepted and always sufficient cause. It has not been thirty years since attention was first called to the association of albuminuria with pregnancy. Next the analogy between puerperal and uræmic convulsions was arrived at. But uræmia is not now recognised as explaining all cases, not even all cases of fetal convulsions.

It is not improbable that there are a number of morbid processes operating conjointly, sometimes one, sometimes another process becoming the most efficient cause. Let us, however, see in what respect the pregnant or puerperal individual differs from the same individual non-pregnant. First, in reference to the circulatory fluid. Physiologists tell us, that the modifications in this consists in a lowered specific gravity, an increased amount of water, an increase in the entire bulk of blood, an increase in white blood corpuscles, an increase in urea and in fibrin, a deficiency in albumen; also, the disturbance in respiration permits the retention in the blood of more than the normal amount of carbonic acid. There is, in other words, a plethora, a fullness of the entire vascular system, but a plethora of hydræmic blood, with a deficiency in those elements destined for nutrition, and a superabundance of these elements representative of retrograde tissues metamorphosed as urea and fibrin and carbonic acid. Associated with blood alteration, there is an increase in the propelling force of the heart; an increased vascular tension incident to a hypertrophied condition of the walls of the left ventricle; this hypertrophy being an accompaniment of probably every case of advanced pregnancy. This vascular tension is, of course, greatly increased during the powerful muscular efforts of delivery. Again, the nervous system undergoes a modification apart from its disturbed nutrition. This entire system is doubtless brought into more intimate relationship with the uterine system; especially is this the case, if, as Mr. Lee states, "There are ganglia developed in the gravid uterus, ganglia, which cannot be discovered in the non-pregnant womb." Let us add to these the mechanical interference with the abdominal circulation occasioned by the enlarged uterus. These various conditions are sufficient to occasion convulsions, especially if decided in degree, but more especially, if more immediately determining causes are co-operating. The low specific gravity of the blood, its increased amount, and the increased force with which it is driven through the vessels, all co-operate in greatly increasing the process of exosmosis. This is evinced in the tendency to general anasarca. The intra-cranial tissues partake of this general condition, and there is at first an engorged state of their blood-vessels, then, by the increased exosmosis, an œdema of the brain tissue occurs. If this œdema is marked at the central portion, or the base of the brain, then convulsions may be occasioned, especially if to this state of œdema is added an irritable condition of the brain centre incident to perverted nutrition, and if, also, this increased intrinsic irritability is agravated by irritation reflected from the peculiarly sensitive uterine nerves, or even if this reflected irritation originates in some part not included in the generative organs, or it may be that emotional or atmospheric disturbances become the superadded cause.

But ninety per cent. of cases of puerperal convulsions are associated with albuminuria. The result is an aggravation of that condition of the system which exists in the pregnant woman without albuminuria. There is further decrease in the highly nutritious elements of the blood's specific gravity, a further increase in the already abnormal amount of urea. Uræmia becomes now the most active agent in the causation of the convulsions, but it is not acting alone, it is only the most prominent of the different etiological conditions; the albuminuria is itself, probably, but the result mainly of the disturbance in the relationships of the nutritive functions, a disturbance incident to the altered character of the circulatory fluid. Whether the increased amount of fibrine is directly or indirectly concerned in the production of the convulsions, has not, as far as I have been able to ascertain, been considered by writers on this subject; yet if we accept as conclusive the investigations of recent physiologists, fibrine like urea is a representative of broken down tissue, and is an effete material, destined to be eliminated from the system. If effete, its presence in the blood in abnormal amounts must prove deleterious, and may probably be concerned, indirectly or directly, in the production of convulsions. Further pathology must determine this point for us.

In addition to the altered state of the blood that I have already referred to, must be included, as an additional cause, septicæmia and pyæmia.

In my experience of eclampsia in the puerperal state, I have observed the following facts:

1. There has always been albuminuria, or, more strictly speaking, uræmia, although undiscovered, from lack of thoroughness in examination.

2. In a majority, the convulsive seizures occur between the fifth and seventh months of utero-gestation.

3. That a firm fibre with an a diposetemperament are the class *a priori*, in which we would most frequently expect convulsions.

4. When setting in after labor, with complete, or nearly complete, suppression of urine, death is inevitable.

5. As a nearly exceptionless rule, when labor has declared itself, empty the uterus as soon as practicable, no matter at what period of pregnancy occurring. .

6. When there is no declared action of the womb, refrain from interference, unless at full term, when, if convulsions persist, empty the uterus.

7. Almost always, when occurring prior to full term, the life of the fœtus is destroyed; occasionally this happens at full term: these results being due to convulsions, or blood-poisoning to the fœtus, or both.

8. When conditions are favorable, extract blood largely.

9. Chloroform must always be an adjuvant to venesection, or, when the former is not practicable, give to control fits.

10. The use of some free evacuant to bowels, skin and kidneys is most imperatively demanded, the object being, as far as possible, to unload the blood of the poisonous principles—the elements of the urine.

There is no more frightful accident that can befall the parturient woman than eclampsia. It is a moment when the weakness of humanity cries loudest for aid. All around are in confusion and terror, while none but the accoucheur himself is cool and self-possessed, and it is only experience and an innate consciousness of his ability to cope with the malady that can give him a steady hand.

The peculiar interest that has always invested diseases of marked fatality is nowhere more conspicious than in the subject under consideration. Its importance, together with a successful issue of the case, I hope will be deemed a sufficient apology for this paper.

